# Influence of energy drinks on enamel erosion: *In vitro* study using different assessment techniques

**DOI:** 10.4317/jced.57788

**Published:** 2021-11-01

**Authors:** José-Gabriel-Victor-Costa Silva, João-Paulo-Gomes Martins, Elizabeth-Barreto-Galvão de Sousa, Nayanna-Lana-Soares Fernandes, Ingrid-Andrade Meira, Fábio-Correia Sampaio, Andressa-Feitosa-Bezerra de Oliveira, Ana-Maria-Barros-Chaves Pereira

**Affiliations:** 1 Undergraduate Dentistry Course, Health Sciences Centre, Federal University of Paraíba-UFPB, João Pessoa/Paraíba, Brazil; 2 Department of Clinical and Social Dentistry, Federal University of Paraíba-UFPB, João Pessoa/Paraíba, Brazil; 3 Department of Prosthodontics and Periodontology, Piracicaba Dental School, University of Campinas- UNICAMP, Piracicaba/São Paulo, Brazil; 4 Department of Morphology, Federal University of Paraíba-UFPB, João Pessoa/Paraíba, Brazil

## Abstract

**Background:**

Dental erosion has become a relevant public health problem in recent years and is related to the increase in the consumption of acidic beverages. Objective: The aim of the present study was to evaluate the erosive potential of energy drinks on dental enamel using an in vitro erosion model.

**Material and Methods:**

Thirty-eight blocks of human enamel were divided into four groups: G1- TNT Energy Drink®(n=8), G2- Red Bull® (n=10), G3- Monster Energy® (n=10), and G4- Coca-Cola® (n=10) (positive control). For the chemical analysis, the pH values, titratable acidity, and buffering capacity of the beverages were measured in triplicate. For the erosive test, the specimens were immersed in the beverages (5ml/block) for 30 minutes at room temperature with gentle shaking. Initial and final surface microhardness values were measured and the percentage of the loss of surface microhardness was calculated. Profilometry (surface loss and lesion depth) and mineral loss analysis (quantitative light-induced fluorescence) were performed. The data were analysed statistically using ANOVA followed by the Bonferroni correction, Pearson’s correlation test, and multiple linear regression (*p*<0.05).

**Results:**

The energy drinks had pH values ranging from 2.36 to 3.41. The lowest titratable acidity value was recorded for Monster Energy® and the highest was recorded for TNT Energy Drink®. All energy drinks had buffering capacity values higher than Coca-Cola®. Analysing the eroded enamel surface, the specimens submitted to TNT Energy Drink® had the greatest percentage loss of surface microhardness, surface loss, depth, and mineral loss, followed by those submitted to Red Bull® and Monster Energy®. Surface loss was the only predictor of mineral loss (*p*<0.001).

**Conclusions:**

Based on the study model employed, all the energy drinks examined were erosive to tooth enamel and TNT Energy Drink® had the worst behaviour.

** Key words:**Energy drinks, tooth erosion, tooth demineralisation, hardness tests, quantitative light-induced fluorescence.

## Introduction

Dental erosion is a non-bacterial chemical process that leads to the softening and cumulative loss of hard dental tissues (1). Aetiologically, it is associated with the frequent exposure of these tissues to acids, which can be of an intrinsic or extrinsic origin (2,3). Acids of an intrinsic origin are derived from eating disorders, gastroesophageal reflux, and a high frequency of vomiting. The main source of extrinsic acid exposure is a diet rich in acidic foods and beverages (3).

According to Gambon, Brand, and Veerman (4), changes in the dietary habits of populations in recent years, involving an increase in the consumption of acidic beverages, are associated with the higher incidence of dental erosion. Epidemiological studies demonstrate that adolescents and young adults in Brazil and other countries (5,6) are highly affected, with the risk of enamel erosion twice as high in males (6). Such evidence shows that dental erosion has become a relevant public health problem in recent years and, therefore, early diagnosis is essential to the treatment and monitoring of the condition (1,7,8).

Among the diverse processed beverages available on the market, a significant increase in the consumption of energy drinks has occurred in recent years, exceeding 5.8 billion litres in 160 countries in 2013 (9). Such beverages emerged to provide an increase in energy due to their caffeine, taurine, and sugar content (9,10). Thus, the attraction and consumption of energy drinks is greater among young individuals, especially university students, due to both the pleasant taste and the promise of being a stimulant that keeps them awake (10). Lussi *et al*. (11), Saads Carvalho and Lussi (3), and Cavalcanti *et al*. (12) report that energy drinks have low pH values, characterising them as acidic beverages.

The chemical characteristics of a beverage, such as pH, acid titration, buffering capacity, type of acid, as well as the presence of calcium, fluorine, and phosphate, are important factors to measure for the determination of erosive potential (3,11,13). Moreover, studies have used surface microhardness and profilometry to quantify and monitor the degree of softening of the eroded enamel surface as well as both surface and volumetric loss in initial erosive lesions (14,15). Quantitative light-induced fluorescence (QLF) has also gained support and validity in the study of dental erosion (14,16), as it enables the non-destructive determination of the degree of demineralisation. However, no studies were found in the literature involving the *in vitro* comparison of QLF to microhardness and profilometric analysis.

In view of the need for a more accurate assessment of the surface topography of tooth enamel exposed to acidic beverages, the aim of the present study was to evaluate the erosive potential of energy drinks on dental enamel subjected to an erosive challenge, associating chemical factors with microhardness, profilometric, and quantitative light-induced fluorescence (QLF) techniques. The null hypothesis is that there are no surface changes on enamel subjected to an erosive challenge with energy drinks using different methods of analysis.

## Material and Methods

-Ethical considerations

This study received approval from a research ethics committee in Brazil (certificate number: 45917915.6.0000.5188). The donors of the teeth signed a statement of informed consent in accordance with the Declaration of Helsinki and Resolution 466/12 of the Brazilian National Health Board.

-pH, titratable acidity, and buffering capacity of beverages 

Three commercially popular energy drinks were selected and purchased for analysis (Table 1). The soft drink Coca-Cola® was used as the positive control, as its high erosive potential is already known in the scientific literature (3,17). All beverages were stored according to the manufacturer’s instructions.

**Table 1:**
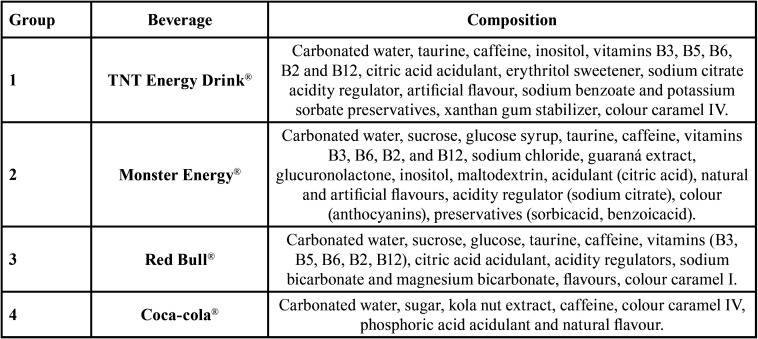
Compositions of the experimental beverages as listed on their respective packaging.

Immediately after opening each beverage, pH and titratable acidity (TA) were measured in triplicate at room temperature. The pH was measured using a previously calibrated pH meter (Orion 290A+, Thermo Electron Corporation) with the substance placed in a beaker and stirred using a non-heating magnetic stirrer until a reaching stable reading. TA was determined as the volume of a standard 1M NaOH solution required to increase the pH of 50 mL of each beverage to 5.5 and 7.0. The solution was added at increments of 0.2ml while stirring with a non-heating magnetic stirrer until a stable pH reading was achieved. The pH values were then converted to mmol. Buffering capacity (β) was calculated based on Lussi *et al*. (11) using the following equation: β=ΔC/ΔpH, in which ΔC is the total amount of base used to raise the initial pH to 7.0 and ΔpH is the change in the pH of the solution.

-Preparation of specimens

Thirty-eight enamel specimens (4×4×2 mm) were prepared from extracted human third molars and stored in a 0.08% thymol solution. It was used 10 enamel samples per group based on a study that examined acidic beverages (18). The specimens were embedded in self-curing acrylic resin using circular moulds measuring 16mm diameter and 3mm in depth. The enamel surface was ground flat using sand paper (grits: 600 to 1500) with water cooling and polished with 1 µm diamond paste (Extec Corporation, Enfield, CT) in a rotating polishing machine (PSK-2V, Skill-Tec Comércio e Manutenção Ltda, São Paulo, SP, Brazil).

After 5 min of sonication in a water bath using an ultrasonic device, baseline enamel surface microhardness (SH0) was determined using a microhardness tester (Shimadzu HMV - AD Easy Test Version 3.0). Three indentations spaced 100µm apart were made at the centre of the enamel surface (Vickers, 100g, 10s). The upper and lower portions of the exposed enamel surface were covered with two layers of nail varnish (Risqué, Niasi, Taboão da Serra, São Paulo, Brazil) as a reference area for the surface microhardness, profilometry, and QLF analysis. The central portion (1 mm) remained uncovered for the erosion assay.

-Erosive challenge

For the induction of erosion, the samples in each group were immersed in 50 ml of the substances (5 ml of beverage/mm2 of exposed enamel specimen) at room temperature (22-25ºC) for 30 min with gentle stirring. The specimens were then rinsed with deionised water and stored in a humidity-controlled environment to prevent drying until further analysis.

**Figure 1:**
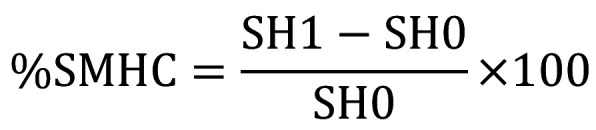
Formula.

After acid exposure, the nail varnish was carefully removed using an acetone solution (1:1 water) and post-demineralisation surface hardness (SH1) was determined using the same procedures described above. The percentage of surface hardness change was calculated as follows: (Fig. 1).

-Analysis of enamel surface loss and roughness 

Three-dimensional non-contact profilometry (TALYSURF CCI MP, Taylor Hobson, England) was used with a 0.25-mm cut-off, 20× lens, numerical aperture of 0.4, and 1× scan speed in the “XYZ” mode to measure the loss of enamel surface area (Sa, µm) and lesion depth (“step”). An area of approximately 0.86×0.86 mm (0.74 mm2) was scanned, covering the treated and reference surfaces. To determine the loss of enamel surface area (Sa, µm), a rectangle of sound and eroded regions was selected and a surface area loss value was obtained as the difference between the sound and eroded surfaces. To calculate lesion depth (“step”), linear tracings were considered at three different levels of the sample: 75% (upper third), 50% (middle third), and 25% (lower third). At each level, the step was calculated by subtracting the height of the eroded area from the height of the sound reference surface. The arithmetic mean of the three levels was used. Surface profilometric images were obtained and used for the qualitative analysis of the surface finish obtained with each energy drink and Coca-Cola®.

-Quantitative fluorescence analysis 

The enamel blocks were evaluated for fluorescence loss in the erosion lesions compared to sound areas. The samples were dried prior to analysis. To standardise the quantitative light-induced fluorescence (QLF) measurements, the camera was attached to a tripod in the same position for all images. The specimens were fitted to the Qraycam pro device with an exposure of 0, contrast of 0, and a distance between the device and sample of 8 cm (following the manufacturer’s instructions) in a dark room. All images were analysed using the Q-ray software (version 1.38, Inspektor Research Systems) to quantify changes in fluorescence intensity compared to the sound enamel surface based on the ΔFmax value, which is the percentage decrease in autofluorescence intensity in an erosion lesion compared to that of sound enamel and reflects changes in the mineral content of the enamel (19).

-Statistical analysis

The data were analysed statistically using the SPSS package for Windows, version 21.0 (SPSS, Inc., Chicago, IL, USA). The Shapiro-Wilk test and Levene’s test were used to determine normality and homogeneity of variances, respectively. As the data demonstrated equal variances and Gaussian distribution, no data transformation was needed. The following tests were performed: 1) ANOVA with the Bonferroni correction for the analysis of differences between groups regarding pH, TA, buffering capacity, SH0, SH1, %SMHC, surface loss, step, and ΔFmax; 2) Pearson’s correlation; and 3) multiple linear regression with adjusted r² values and a 5% significance level. The quantification of significant associations between mineral loss (ΔFmax) and the independent variables (surface loss, step and %SMHC) was adjusted in the regression model using the association at the 30% level. The model was run using the backward elimination procedure, with the removal of unimportant variables one-by-one and the adjustment of the model to the most relevant variables. Through the model, the parameters and risk values for each variable were estimated. The confidence level was 5%.

## Results

The present study evaluated the erosive potential of three energy drinks and Coca-Cola® (positive control) using chemical and quantitative analyses of surface hardness, roughness, and mineral loss.

Table 2 displays the mean pH, titraTable acidity (TA) for pH 5.5 and 7.0, and buffering capacity (β) values. All energy drinks evaluated had pH lower than 7, characterizing acidic beverages. TNT Energy Drink® had the lowest pH value. Statistically significant differences were found between two of the energy drinks and the positive control (Coca-Cola®). The exception was TNT Energy Drink® (*p*>0.05).

**Table 2:**
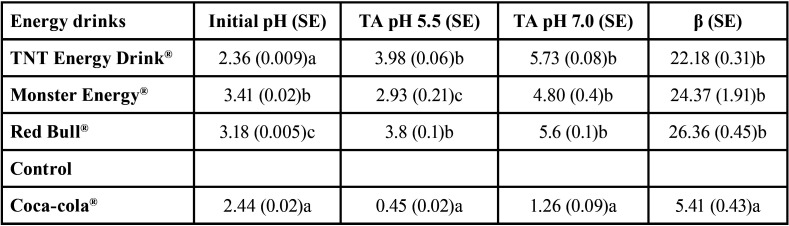
Average of initial pH values, Titratable Acidity (TA), for pHs 5.5 and 7.0, and Buffering Capacity (β) of allanalyzed drinks.”

Regarding TA, TNT Energy Drink® needed a greater amount of base to reach pH values 5.5 and 7.0, followed by Red Bull® and Monster Energy®. All energy drinks had higher buffering capacity values compared to Coca-Cola®. Based on the chemical parameters investigated, the energy drinks had greater erosive potential than Coca-Cola®. Moreover, significant correlations were found between the pH and β (r=0.612; *p*<0.05) and between TA for pH 7.0 and β (r=0.960; *p*<0.001).

**Figure 2:**
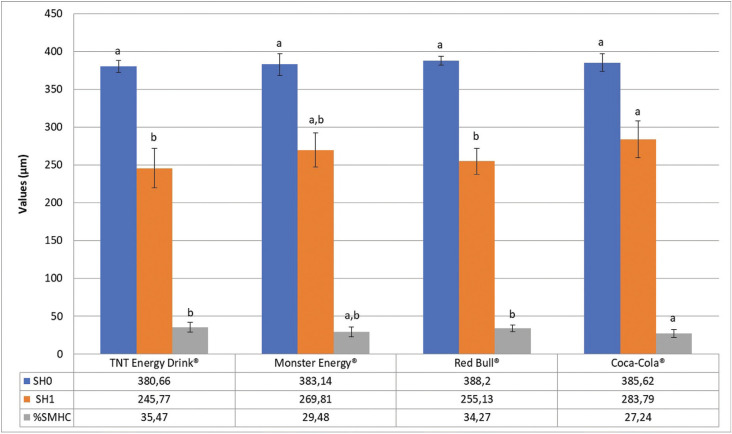
Mean and standard deviation of the values obtained by the initial (SH0) and final (SH1) surface microhardness, in addition to the percentage of loss of surface microhardness (%SMHC) of all groups analyzed. Similar lower case letters, for each variable studied separately (line), represent absence of significance between the groups (ANOVA, *p*>0.05).

Figure 2 displays the mean surface microhardness values. No significant differences among groups were found regarding SH0, demonstrating the uniformity and standardisation of the samples of sound enamel. In contrast, significant differences among groups were found regarding SH1. Monster Energy® did not differ statistically from Coca-Cola® (*p*>0.05) for SH1. The %SMHC value was highest for TNT Energy Drink®, followed by Red Bull® and Monster Energy®. Significant differences were found between Coca-Cola® and both TNT Energy Drink® and Red Bull® (*p*<0.05).

**Figure 3:**
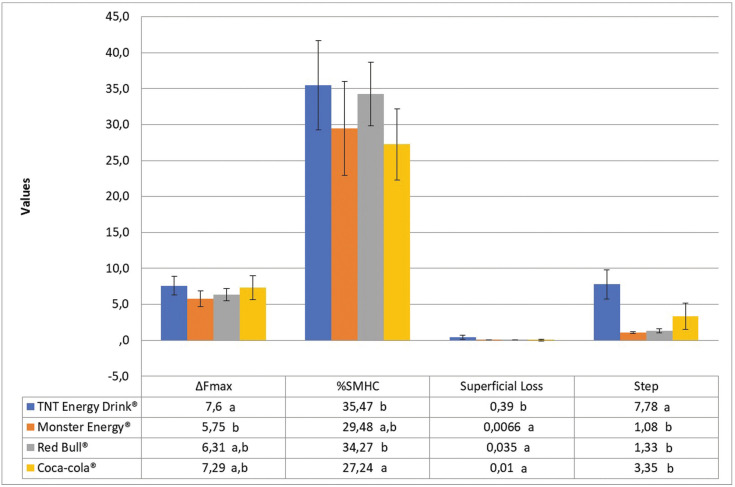
Mean and standard deviation of values obtained by mineral loss (ΔFmax), Superficial Loss, Step and %SMHC of all analyzed groups (The negative values obtained for ΔFmax and Superficial Loss were multiplied by -1 for presentation in the figure). Similar lower case letters, for each variable studied separately (column), represent absence of significance between groups (ANOVA, *p*>0.05).

Figure 3 displays the results of the profilometric analysis of the enamel surface. All drinks led to an increase in surface roughness and depth (step) of the lesion. TNT Energy Drink® had the highest values for these variables, following the same pattern as that found in the analysis of the amount of mineral loss by fluorescence (ΔFmax) and %SMHC. The QLF analysis revealed no significant difference in mineral loss in the comparison of the energy drinks to the positive control (Coca-Cola®) (*p*>0.05).

Significant correlations were found between surface loss and mineral loss (r=0.500; *p*=0.001), between step and mineral loss (r=0.478; *p*=0.002), and between pH and step (r=-0.793; *p*=0.002).

Surface loss (β=0.500; t=3.461; *p*<0.001) was the only predictor of mineral loss (ΔFmax) according to the statistically significant model [F(1.36)=11.98; *p*<0.001; R2=0.250] (Table 3).

**Table 3:**
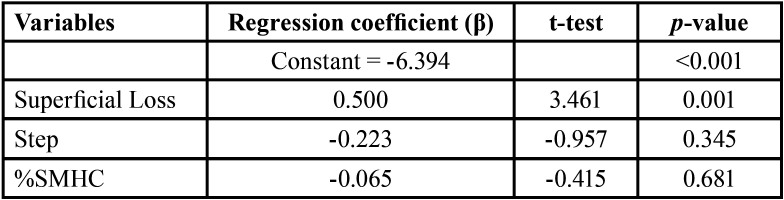
Multiple linear regression analysis for mineral loss (ΔFmax), obtained with QLF, and its independent variables.

## Discussion

The exposure of teeth to acidic substances causes changes in the structural integrity and physical properties of the dental structure, resulting in the softening and subsequent loss of tissue (20). Thus, it is important to know the erosive potential of acidic beverages. Under normal conditions, the enamel is constantly exposed to an acidic environment and its demineralisation depends on the pH and concentration of mineral ions in the surrounding fluids (21,22). However, there is no critical pH for enamel erosion. The erosive potential of each beverage depends on its Ca2+ and phosphate content (23).

The energy drinks evaluated in the present study tended to higher pH values than Coca-Cola®, which is in agreement with data reported in previous studies (3,11,12,18,24). However, these are potentially erosive drinks, especially if consumed quite often. Citric acid is often found in the composition of energy drinks and is highly erosive, exerting a demineralising effect on enamel even after the pH has been neutralised (25). Moreover, both titratable acidity and buffer capacity are closely related to pH and higher values are associated with erosive potential (3,11,22).

Enamel is affected as the dissolution rate increases and the pH decreases (20,21,22). Different methods of analysis are recommended for the study of dental erosion (14,15,16,26). However, each tool has its limitations and the use of only one of these methods does not furnish a full understanding of the changes that have occurred on the surface of tooth enamel (15).

The %SMHC values of the energy drinks revealed a reduction in surface microhardness after the erosive challenge, which is in agreement with data reported by Lussi & Carvalho (20). TNT Energy Drink® and Red Bull® had the highest %SMHC values, which differed significantly from that of the positive control (Coca-Cola®) (*p*<0.05). The literature reports a greater loss of surface microhardness with the increase in exposure time, especially when combined with vigorous agitation (20). In the present study, the enamel specimens could have been more severely compromised if they had been exposed to the acid challenge for a longer period (27). Following the same pattern as that found for all other variables, TNT Energy Drink® presented the highest and statistically significant values regarding surface loss and lesion depth among all energy drinks tested. The profilometer may also detect higher values with the increase in exposure time (14). Therefore, it is important to collect data on the time and frequency of exposure for the prevention and treatment of dental erosion (1,2,3,12).

QLF has proven effectiveness in the quantification of erosive lesions. The evaluation of dental erosion is based on the property of enamel autofluorescence, which is reduced with the mineral loss caused by erosion (14,16,28). In the present study, all beverages tested led to changes in enamel autofluorescence, reflecting mineral loss and revealing the erosive potential of these beverages. No previous studies were found in the literature describing QLF analysis for the detection of mineral loss from energy drinks. QLF analysis is comparable to transverse microradiography (TMR), which, although considered the “gold standard”, has the disadvantage of being a destructive technique (14,15). Therefore, recent studies addressing eroded enamel have encouraged the use of QLF (28). In the linear regression model, surface loss was the predictor of ΔFmax, confirming that the profilometer is a good tool for studying enamel erosion (15). Moreover, pH was significantly correlated with both ΔFmax and lesion depth, which confirms the altered surface integrity of enamel when attacked by acids, resulting in a vulnerable softened layer with subsequent tissue loss (3,8,12,29).

Besides the harm to enamel due to frequent consumption (10), energy drinks have a number of ingredients that exert negative effects on health (9,24). The preventive management of dental erosion is quite complex due to the multifactor aetiology and the involvement of nutritional and individual aspects, culminating in the progression of erosive lesions (2,3). Therefore, patients should be instructed regarding the potential harmful effects of such beverages when consumed frequently (12).

It should be emphasized that the present study followed an *in vitro* design. Thus, the results cannot be fully transferred to the clinical reality, once biological factors involving saliva and its protective effect, in addition to the quantity, time, and frequency of beverages consumption can influence the *in vivo* erosion progression (26,30).However, due to the high incidence of dental erosion, there is a great need for standardized protocols and studies that accurately reproduce the erosive process *in vitro*, to facilitate its understanding on dental tissues (30) .

Finally, the present investigation satisfies the need described by Attin and Wegehaupt (26) for more detailed studies on surface changes in cases of dental erosion using different methods of analysis (15). All energy drinks tested were erosive, as tissue changes were found in all enamel samples with the different analysis methods employed. The TNT Energy Drink® had the greatest erosive potential, as demonstrated by the high %SMHC, surface loss, lesion depth, and ΔFmax values.
